# Dissimilatory Metabolism of Nitrogen Oxides in Bacteria: Comparative Reconstruction of Transcriptional Networks

**DOI:** 10.1371/journal.pcbi.0010055

**Published:** 2005-10-28

**Authors:** Dmitry A Rodionov, Inna L Dubchak, Adam P Arkin, Eric J Alm, Mikhail S Gelfand

**Affiliations:** 1 Institute for Information Transmission Problems, Russian Academy of Sciences, Moscow, Russia; 2 Genomics Division, Lawrence Berkeley National Laboratory, Berkeley, California, United States of America; 3 Physical Biosciences Division, Lawrence Berkeley National Laboratory, Berkeley, California, United States of America; 4 State Scientific Center GosNIIGenetika, Moscow, Russia; 5 Department of Bioengineering and Bioinformatics, Moscow State University, Moscow, Russia; University of Tokyo, Japan

## Abstract

Bacterial response to nitric oxide (NO) is of major importance since NO is an obligatory intermediate of the nitrogen cycle. Transcriptional regulation of the dissimilatory nitric oxides metabolism in bacteria is diverse and involves FNR-like transcription factors HcpR, DNR, and NnrR; two-component systems NarXL and NarQP; NO-responsive activator NorR; and nitrite-sensitive repressor NsrR. Using comparative genomics approaches, we predict DNA-binding motifs for these transcriptional factors and describe corresponding regulons in available bacterial genomes. Within the FNR family of regulators, we observed a correlation of two specificity-determining amino acids and contacting bases in corresponding DNA recognition motif. Highly conserved regulon HcpR for the hybrid cluster protein and some other redox enzymes is present in diverse anaerobic bacteria, including Clostridia, Thermotogales, and delta-proteobacteria. NnrR and DNR control denitrification in alpha- and beta-proteobacteria, respectively. Sigma-54-dependent NorR regulon found in some gamma- and beta-proteobacteria contains various enzymes involved in the NO detoxification. Repressor NsrR, which was previously known to control only nitrite reductase operon in *Nitrosomonas* spp., appears to be the master regulator of the nitric oxides' metabolism, not only in most gamma- and beta-proteobacteria (including well-studied species such as *Escherichia coli*), but also in Gram-positive *Bacillus* and *Streptomyces* species. Positional analysis and comparison of regulatory regions of NO detoxification genes allows us to propose the candidate NsrR-binding motif. The most conserved member of the predicted NsrR regulon is the NO-detoxifying flavohemoglobin Hmp. In enterobacteria, the regulon also includes two nitrite-responsive loci, *nipAB (hcp-hcr)* and *nipC (dnrN),* thus confirming the identity of the effector, i.e. nitrite. The proposed NsrR regulons in *Neisseria* and some other species are extended to include denitrification genes. As the result, we demonstrate considerable interconnection between various nitrogen-oxides-responsive regulatory systems for the denitrification and NO detoxification genes and evolutionary plasticity of this transcriptional network.

## Synopsis

Comparative genomics is the analysis and comparison of genomes from different species. More then 100 complete genomes of bacteria are now available. Comparative analysis of binding sites for transcriptional regulators is a powerful approach for functional gene annotation. Knowledge of transcriptional regulatory networks is essential for understanding cellular processes in bacteria. The global nitrogen cycle includes interconversion of nitrogen oxides between a number of redox states. Despite the importance of bacterial nitrogen oxides' metabolism for ecology and medicine, our understanding of their regulation is limited. In this study, the researchers have applied comparative genomic approaches to describe a regulatory network of genes involved in the nitrogen oxides' metabolism in bacteria. The described regulatory network involves five nitric oxide−responsive transcription factors with different DNA recognition motifs. Different combinations of these regulators appear to regulate expression of dozens of genes involved in nitric oxide detoxification and denitrification. The reconstructed network demonstrates considerable interconnection and evolutionary plasticity. Not only are genes shuffled between regulons in different genomes, but there is also considerable interaction between regulators. Overall, the system seems to be quite conserved; however, many regulatory interactions in the identified core regulatory network are taxon-specific. This study demonstrates the power of comparative genomics in the analysis of complex regulatory networks and their evolution.

## Introduction

**Figure 1:**
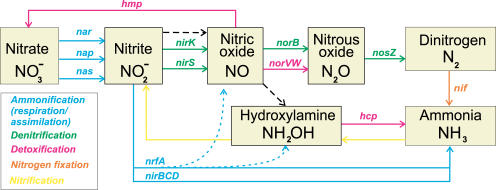
The Bacterial Inorganic Nitrogen Cycle The ammonification, denitrification, detoxification, nitrogen fixation, and nitrification pathways are shown by colored solid lines with genes names involved in the pathway. The dashed black line shows possible non-enzymatic interconversions of nitrogen oxides. The dotted line shows additional formation of NO and hydroxylamine during nitrite ammonification.

Interconversion of nitrogen species between a number of redox states forms the biogeochemical nitrogen cycle, which has multiple environmental impacts and industrial applications. Bacteria can utilize soluble nitrogen oxides, nitrate and nitrite, as terminal electron acceptors in oxygen-limiting conditions. Two dissimilar pathways of nitrate respiration, ammonification and denitrification, involve formation of a common intermediate, nitrite, but end in different products, ammonia and gaseous nitrogen oxides or dinitrogen, respectively (Figure 1). At the first step, nitrite is formed by one of three different types of nitrate reductases: soluble assimilatory Nas, membrane-associated respiratory Nar, and periplasmic dissimilatory Nap. The next step of ammonification is conversion of nitrite into ammonia by either respiratory cytochrome *c* nitrite reductase NrfA or detoxifying siroheme-containing enzyme NirBD [1]. In contrast, during denitrification, nitrite is reduced to nitric oxide (NO), nitrous oxide, and, finally, dinitrogen, using nitrogen oxide reductases NirK (or NirS), NorB, and NosZ, respectively [2].

NO is a signaling and defense molecule in animals, but bacteria are sensitive to high NO concentrations due to its reactivity and membrane permeability [3]. NO and hydroxylamine, two toxic intermediates in 6-electron reduction of nitrite, could be formed during nitrite ammonification [4,5]. In addition to a classical NO reductase (NorB) present in denitrifying species, two other bacterial NO detoxification enzymes have been characterized: an NO reductase (flavorubredoxin NorVW in *Escherichia coli*) [6] and an NO dioxygenase (flavohemoglobin Hmp or Fhp in *E. coli, Bacillus subtilis, Ralstonia eutropha,* and *Pseudomonas* species) [7–9].

An unusual redox enzyme, called the hybrid cluster protein (Hcp) or “prismane protein,” has been extensively studied in strictly anaerobic (*Desulfovibrio* species) and facultative anaerobic *(E. coli, Salmonella typhimurium, Acidothiobacillus ferrooxidans, Shewanella oneidensis)* bacteria, where it is induced mostly during conditions of nitrite or nitrate stress, suggesting a role in nitrogen metabolism [10–14]. In the latter bacteria, the *hcp* gene always forms a possible operon with NADH oxidoreductase *hcr,* whose product catalyzes reduction of Hcp in the presence of NADH [11]. Until recently, the in vivo electron-accepting substrate of Hcp was unknown, and based on the crystal structure, NO was assumed to be a good candidate for this role [12,15]. In vitro studies demonstrated oxygen-sensitive hydroxylamine reductase activity of the *E. coli* Hcp protein, suggesting its possible role in detoxification of reactive by-products of nitrite reduction [16]. More recently, the requirement of the *hcp* gene for in vivo hydroxylamine reduction was observed in *Rhodobacter capsulatus* E1F1 [17].

Expression of the denitrification genes is known to be activated by nitrogen oxides and low oxygen tension [18]. Both in denitrifying and ammonifying γ-proteobacteria, the nitrate/nitrite signal is processed by the two-component sensor-regulator NarX-NarL and its paralog NarQ-NarP in *E. coli* that control the respiratory nitrate reductase operon *nar* and the nitrite ammonifying loci *nir* and *nrf* [19]. Various transcription factors of the FNR family have been described as NO-sensing regulators of denitrification: DNR in *Pseudomonas* species, NNR in *Paracoccus denitrificans,* and NnrR in *Rhodobacter sphaeroides* and *Bradyrhizobium japonicum* [18]. The DNR/NNR and NnrR proteins cluster phylogenetically in separate subgroups, separately from other family members including FNR, a global regulator of anaerobiosis in facultative anaerobic bacteria [20].

Another NO-responsive transcriptional factor, σ^54^-dependent NorR, activates expression of the NO reductases *norVW* in *E. coli* and *norAB* in *R. eutropha* [21,22]. Three tandem upstream activator sites with the core consensus GT-(N_7_)-AC were identified as NorR-binding sites observed in both promoter regions. Analysis of the adjacent regions of additional *norR* orthologs in bacterial genomes revealed similar tandem NorR-binding sites upstream of the *norA* and *norB* genes in *Ralstonia* species, *norVW* in *Salmonella* species, *hmp* in *Vibrio cholerae* and *Pseudomonas aeruginosa,* and *hcp* in *V. vulnificus* [23].

A nitrite-sensitive transcriptional repressor, named NsrR, has been identified in lithoautotrophic β-proteobacterium *Nitrosomonas europeae,* where it regulates expression of the copper-nitrite reductase *nirK* [24]. Co-localization of the *nsrR* ortholog and the *hcp* gene in *R. capsulatus* E1F1 suggested that NsrR and nitrite could be involved in the regulation of hydroxylamine assimilation in this α-proteobacterium [17]. NsrR is a member of the Rrf2 family of transcriptional regulators, which includes a putative Rrf2 regulator for a redox operon in *D. vulgaris* [25], the iron-responsive repressor RirA, which controls iron uptake in rhizobia [26], and the IscR repressor for the Fe-S cluster assembly operon in *E. coli* [27].

Despite this diversity of regulatory systems, our understanding of the regulation of the nitrogen oxides metabolism in bacteria is very limited. For example, NO- and nitrite-dependent activation of expression of *hmp* in *E. coli* and *B. subtilis, hcp-hcr (nipAB)* and *dnrN (nipC)* in *S. typhimurium,* and *norB* and *aniA (nirK)* in *Neisseria gonorrhoeae* has been described [28–30], but specific transcriptional factors involved in this control are not yet known. In this study, we analyzed regulation of the nitrosative stress and denitrification genes in available bacterial genomes using comparative genomics approaches [31,32] and predicted a large number of new regulatory elements for these genes. In addition to a complete description of the previously known NorR, DNR, and NnrR regulons, we report identification of a novel FNR-like regulator, named HcpR, for the *hcp* and other redox-related genes in anaerobic bacteria. Starting from very limited data, we were able to identify the NsrR-binding motif and describe the NsrR regulons in sequenced γ- and β-proteobacteria, as well as in the *Bacillus* and *Streptomyces* species. Combining published experimental and newly obtained comparative data, we have reconstructed the NO- and nitrite-dependent transcriptional regulatory network for dissimilatory metabolism of nitrogen oxides in bacteria.

## Results

### HcpR: Recognition Motifs and Core Regulon

A member of the CRP/FNR family of transcription factors, HcpR has been initially identified as the regulator of the *hcp* gene encoding the hybrid cluster protein and the *frdX* encoding a ferredoxin-like protein in the *Desulfovibrio* species, anaerobic metal-reducing δ-proteobacteria [33]. The consensus of the candidate HcpR binding sites, wTGTGAnnnnnnTCACAw, is similar to the CRP consensus of *E. coli*.

**Figure 2:**
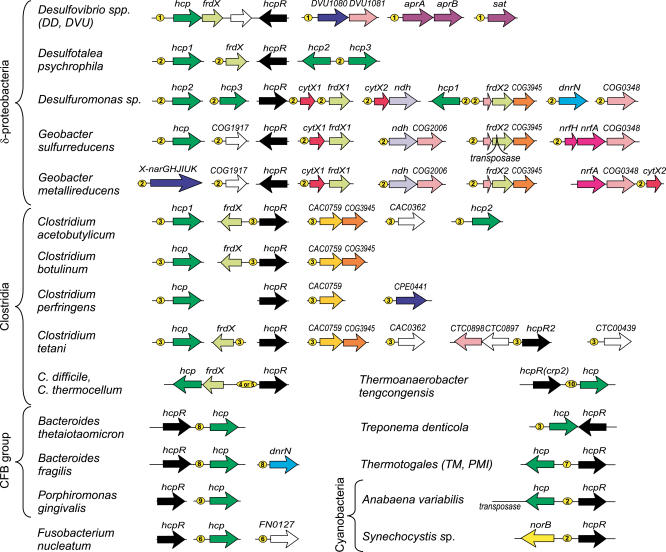
Genomic Organization of Genes Regulated by HcpR Yellow circles with numbers denote candidate HcpR sites with different consensus sequences. These numbers correspond to the HcpR profile numbers in Figure 3.

Close *hcpR* orthologs were detected in other δ-proteobacteria, namely two *Geobacter* species, *Desulfotalea psychrophila* and *Desulfuromonas*. However, the same CRP-like motifs were not present in these genomes. As the analysis of the regulator multiple alignment revealed a substitution in the helix-turn-helix motif involved in DNA recognition that could cause this change (see “Co-evolution of regulators and their recognition motifs” for details), and since the considered species have multiple *hcp* and *frdX* paralogs, we applied the motif detection procedure to a set of corresponding upstream regions and obtained a new FNR-like palindromic motif with consensus sequence wyTTGACnnnnGTCAArw, which has a notable distinction from the CRP-like motif in the third position (not G, but T). This recognition motif was observed upstream of most *hcp* and *frdX* paralogs in the studied δ-proteobacteria, as well as upstream of some additional genes in *Desulfuromonas* and *Geobacter* species (Figure 2 and Table S1).

**Figure 3:**
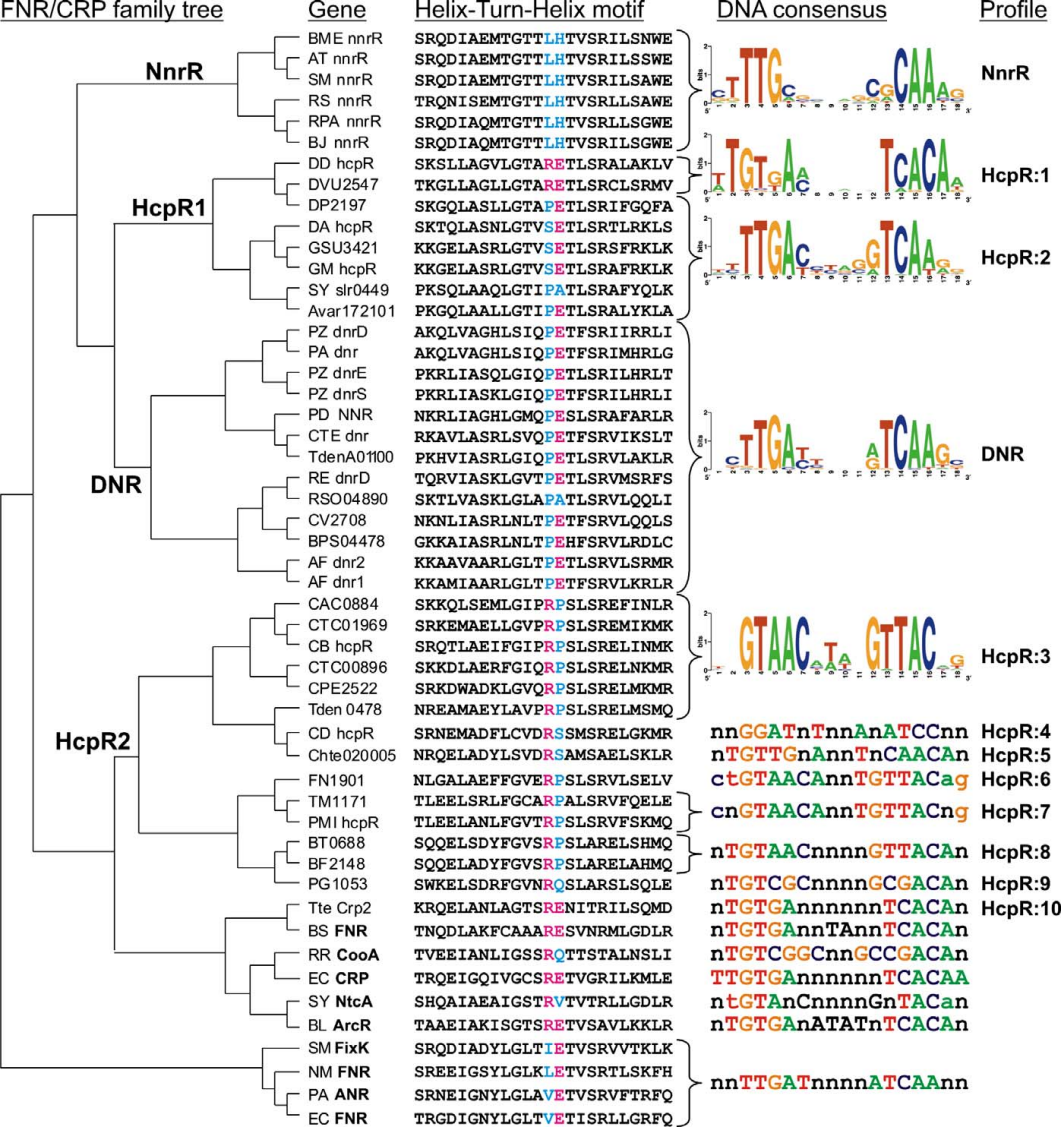
Maximum Likelihood Phylogenetic Tree of the FNR/CRP Family of Transcriptional Regulators The third column contains sequences of helix-turn-helix motifs in the proteins. Two specificity-determining positions correlated with DNA motifs are colored (R_180_ and E_181_ in proteins correlate with G_3_ and A_6_ in DNA sites, respectively). The fourth column includes sequence logos for presumably homogeneous and large site sets and sequence consensi for small sets of DNA sites and for well-established motifs of other factors (FNR, CRP, CooA, NtcA, ArcR). The last column indicates the name of a search profile constructed in this study.

Close orthologs of *hcpR* from δ-proteobacteria are present in two cyanobacteria, *Anabaena variabilis* and *Synechocystis* sp. (*Avar17201* and *slr0449* in Figure 3), where they are divergently transcribed with the *hcp* and *norB* genes, respectively. Both these genes are preceded by candidate HcpR sites with consensus sequence TTGACnnnnGTCAA, and no other similar sites were found in the genomes of these two cyanobacteria (Figure 2).

To analyze possible regulation of the *hcp* genes in other taxonomic groups of bacteria, we considered their gene neighborhoods and found that genes for FNR-like regulators are often co-localized with *hcp* in most *Clostridium* species, *Bacteroides,* Thermotogales, and *Treponema denticola* (Figure 2). On the phylogenetic tree of the FNR/CRP protein family (Figure 3), all such regulators form a separate branch, named HcpR2, and additional representatives of this branch always co-occur with *hcp* genes in bacterial genomes. By applying the motif recognition procedure to a set of *hcp* upstream regions from HcpR2-containing genomes, we identified a conserved DNA motif with consensus GTAACnnnnGTTAC.

Other types of DNA motifs were observed upstream of the *hcp* genes in *Clostridium thermocellum, C. difficile,* and *Porphyromonas gingivalis,* and upstream of the *hcp* gene in *Thermoanaerobacter tengcongensis*. In the latter species the *hcp* gene has a CRP-like regulatory site and is preceded by the *crp2* gene, which is an ortholog of the *B. subtilis fnr* gene, making it likely that *crp2* regulates *hcp* (Figure 2). The predicted HcpR2 regulons in most *Bacteroides* species, *P. gingivalis, Fusobacterium nucleatum, T. denticola,* and Thermotogales contain only *hcp* genes (Figure 2).

### DNR and NnrR Core Regulons

**Figure 4:**
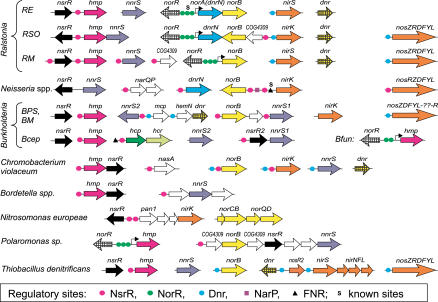
Genomic Organization of Genes Regulated by NsrR, NorR, and DNR in β-Proteobacteria Magenta, green, and blue circles denote candidate NsrR, NorR, and DNR sites, respectively. Candidate σ^54^ promoters associated with NorR sites are shown by angle arrows. Experimentally known sites of NorR and DNR are marked by “s.” Additional sites of the NarP and FNR factors are indicated by purple squares and black triangles, respectively.

In two denitrifying *Pseudomonas* species, *P. stutzeri* and *P. aeruginosa,* expression of the *nir, nor,* and *nos* genes is regulated by the NO-responsive FNR-like transcriptional activator DNR that binds to a DNA motif similar to the consensus FNR box, TTGATnnnnATCAA [34,35]. By a combination of similarity search and phylogenetic analysis of the CRP/FNR protein family (Figure 3), we identified DNR orthologs in the genomes of various denitrifying β-proteobacteria, including three *Ralstonia* and two *Burkholderia* species, *C. violaceum* and *Thiobacillus denitrificans* (Figure 4)*.* To identify the DNR recognition motif in denitrifying species, we selected the upstream regions of denitrification genes encoding nitrite, NO, and nitrous oxide reductases from genomes containing DNR orthologs and applied the motif detection procedure. The resulting FNR box-like motif with consensus CTTGATnnnnATCAAG was identified upstream of most denitrification genes *(nirS, nirK, norB, nosZ)* (Table S2).

**Figure 5:**
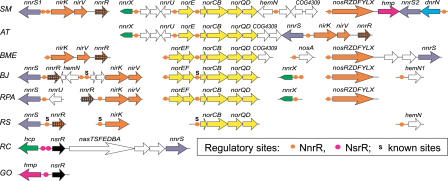
Genomic Organization of Genes Regulated by NnrR and NsrR in α-Proteobacteria Orange and magenta circles denote candidate NnrR and NsrR sites, respectively. Experimentally known NnrR sites are marked by “s.”

No orthologs of the HcpR, DNR, NsrR (below), and NorR (below) regulators were identified in α-proteobacterial genomes. The only exception seems to be *R. capsulatus* E1F1, whose genome contains an *nsrR* ortholog close to the *hcp* gene within the nitrate assimilation *nas* gene cluster [17]. However, in denitrifying species, including *R. sphaeroides* and *B. japonicum,* the FNR-like transcriptional factor NnrR activates expression of nitrite and NO reductases and of the *nnrS* gene [36–38]. Orthologs of *nnrR* were identified in six α-proteobacteria, all of which also possess the *nir* and *nor* genes involved in denitrification (Figure 5). The NnrR orthologs form a separate branch on the phylogenetic tree of the CRP/FNR family (Figure 3). To analyze the NnrR regulon, the motif detection procedure was applied to a training set of the *nir, nor, nos,* and *nnrS* upstream regions from α-proteobacteria. The conserved NnrR recognition motif with consensus ctTTGcgnnnncgCAAag was identified upstream of most denitrification genes (Table S3). The same candidate NnrR sites have been previously identified in *R. sphaeroides* by a comparison of the *nir, nor,* and *nnrS* upstream regions and then confirmed for the latter gene by site-directed mutagenesis [36].

### Co-Evolution of Regulators of the CRP/FNR Family and Their Recognition Motifs

The HcpR recognition motifs identified in several bacteria demonstrated some diversity, which could be correlated with changes in the regulator DNA-binding helix-turn-helix domain. In particular, the CRP-like motif wTGTGAnnnnnnTCACAw of *Desulfovibrio* species differs from the FNR-like motif wyTTGACnnnnGTCAArw in other δ-proteobacteria in the third position (not T, but G). Examination of multiple alignment of the CRP/FNR-like proteins revealed one specific amino acid (R_180_) in the HTH motif involved in DNA recognition, which has changed from arginine (like in *E. coli* CRP and *Desulfovibrio* HcpR) to Ser or Pro in other δ-proteobacteria (see the HcpR1 branch of the phylogenetic tree in Figure 3). Similarly, the difference between the HcpR2 motif GTAACnnnnGTTAC and the motifs of δ-proteobacteria is consistent with substitution of Glu-181 in the DNA recognizing HTH domain to Pro in the HcpR2 proteins (Figure 3).

The structure of CRP in complex with its DNA operator has been determined [39]. Three positions (1ber, chain A residues 180, 181, and 185) interact with the DNA target site, and mutagenesis studies have shown that point mutations at these positions alter the specificity of the protein [40]. We systematically analyzed HcpR, HcpR2, DNR, and NnrR sites identified here, as well as several known consensus sequences for other CRP/FNR-family regulators and observed a correlation of two specificity-determining positions, R_180_ and E_181_, and contacting bases in a DNA recognition motif, G_3_ and A_6_, respectively (Figure 3).

A different substitution is observed in three bacterial species, where position 181 in the HcpR2 protein is filled by either Ser *(Clostridium thermocellum* and *C. difficile),* or Gln *(Porphyromonas gingivalis).* In agreement with these replacements, the candidate HcpR2 motifs in these species differ from the common recognition motif detected for most HcpR2-containing genomes (Figure 3). Finally, the Crp2 regulator in *T. tengcongensis* has CRP-like regulatory motif and is orthologous to the FNR regulator of *B. subtilis.*

The phylogenetic tree of the CRP/FNR regulators (Figure 3) represents four main groups of proteins analyzed in this study: DNR, NnrR, HcpR1, and HcpR2. It also includes several well-studied family members with established DNA-binding consensuses. All respective branches on the tree are deeply rooted, and thus their phylogenetic relationships to each other are not well resolved and differ from results of a more extensive phylogenetic analysis of the CRP/FNR-like transcriptional regulators [41]. Nevertheless, in both trees the HcpR1 and DNR branches cluster together, whereas HcpR1 and HcpR2 form two quite distinct branches on the phylogenetic tree (Figure 3). All these proteins lack the canonical FNR-type cysteine motif, thus excluding their binding of the oxygen-labile Fe-S cluster [41,42].

### NsrR: Recognition Motifs and Core Regulon

The above analysis suggests that HcpR controls the *hcp* genes in strictly anaerobic bacteria. However, a large number of facultative anaerobic bacteria possessing the *hcp* gene lack *hcpR* orthologs. In *E. coli, S. typhimurium,* and *S. oneidensis, hcp* is expressed only under anaerobic conditions in the presence of nitrite or nitrate [10–12]. In an attempt to explain a possible molecular mechanism of this induction, we first aligned the upstream regions of the *hcp* genes from eight enterobacteria and identified two highly conserved regions (Figure S1). The upstream potential recognition motif resembles the consensus sequence of the FNR-binding site and thus most likely is involved in the anaerobic induction of the *hcp-hcr* operon by FNR [12]. The second potential DNA motif, an imperfect inverted repeat with consensus gATGyAT-(N_5_)-ATrCATc located downstream of the FNR site, is likely the binding site for a regulatory protein that responds to nitrogen oxides.

**Figure 6:**
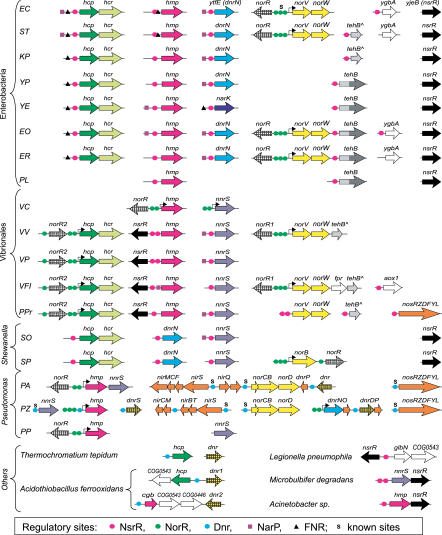
Genomic Organization of Genes Regulated by NsrR, NorR, and DNR in γ-Proteobacteria Magenta, green, and blue circles denote candidate NsrR, NorR, and DNR sites, respectively. Candidate σ^54^ promoters associated with NorR sites are shown by angle arrows. Experimentally known sites of NorR and DNR are marked by “s.” Additional sites of the NarP and FNR factors are indicated by purple squares and black triangles, respectively.

Construction of a recognition rule and search in complete *E. coli* genome identified similar sites in upstream regions of the hypothetical gene *dnrN (ytfE)* and the *hmp* gene encoding NO-detoxifying flavohemoglobin. Importantly, both these candidate sites are highly conserved in multiple alignments of *dnrN* and *hmp* upstream regions from related enterobacteria (Figures S2 and S3). Search in the *S. oneidensis* genome identified the same DNA motif upstream of *hcp-hcr*, *SO4302 (dnrN),* and *SO2805 (nnrS),* the latter encoding a hypothetical heme-copper-containing membrane protein [36]. The *hmp* gene is absent in this genome (Figure 6).

Identification of the conserved palindromic motif suggests that some common transcription factor co-regulates the *hcp-hcr, dnrN, hmp,* and *nnrS* genes in enterobacteria and *Shewanella* species. Since bacterial transcription factors often directly regulate adjacent genes [43], we analyzed gene neighborhoods of genes preceded by the predicted sites (Figures 4 and 6). In many proteobacteria, including Vibrionales, *Acinetobacter* sp., *Chromobacterium violaceum, Ralstonia,* and *Bordetella* species, as well as in Gram-positive bacilli and actinobacteria, the flavohemoglobin gene *hmp* is positionally clustered with a hypothetical transcriptional factor from the Rrf2 protein family. The characterized members of the PF02082 family are the Rrf2 repressor for the electron transport operon *hmc* in *Desulfovibrio vulgaris* [25], the iron-sulfur cluster repressor IscR in *E. coli* [27], the iron-responsive regulator RirA in rhizobia [26], and the nitrite-sensitive repressor NsrR for the nitrite reductase operon *nirK* in *Nitrosomonas europeae* [24]. Phylogenetic analysis (DAR, unpublished data) demonstrated that all representatives of the Rrf2 protein family associated with *hmp* genes appear to be orthologs of the *N. europeae* NsrR protein, and thus we tentatively assign this name to the entire subfamily. Orthologs of the nitrite-sensitive repressor NsrR were identified in all β- and most γ-proteobacteria, being absent only in Pasteurellaceae, Pseudomonadales, and *V. cholerae*. We predict that this transcriptional factor actually binds the identified DNA motif upstream of nitrite/NO-induced genes in enterobacteria and *Shewanella*.

To further analyze the NsrR regulon, we constructed a recognition rule for the NsrR sites and used it to scan the genomes of γ- and β-proteobacteria (Table S4; Figures 4 and 6). The flavohemoglobin gene *hmp* has an upstream NsrR site in most of these genomes, excluding Pseudomonadales, *V. cholerae,* and *Polaromonas* sp., where it is a member of the NO-responsive regulon NorR (see below). The *nnrS* gene, another well-conserved member of the NsrR regulon, was found in some genomes within a possible operon with *nsrR* or *hmp* (Figures 4 and 6). The *norB* gene encoding an NO reductase in denitrifying bacteria is preceded by NsrR sites in the *Neisseria* species, *C. violaceum, Polaromonas* sp., *Ralstonia solanacearum,* and two *Burkholderia* species, *B mallei* and *B. pseudomallei*. Another key enzyme of the denitrification, the copper-containing nitrite reductase NirK, is predicted to be a member of the NsrR regulon in the *Neisseria* species, *C. violaceum,* and *N. europeae* (Figure 4), and in the latter bacterium it was recently shown to be a target of this nitrite-sensitive repressor [24]. In addition to γ-proteobacteria, the *hcp* gene was found under NsrR regulation in a β-proteobacterium *(B. cepacia),* and an α-proteobacterium (*R. capsulatus* E1F1).

Orthologs of *nsrR* have been also found in the complete genomes of most *Bacillus* and *Streptomyces* species, where they are clustered with the flavohemoglobin gene *hmp*. The only exception is *B. subtilis,* which has a stand-alone *nsrR* ortholog, *yhdE*. The predicted NsrR-binding motif appears to be well conserved in these Gram-positive bacteria, and candidate sites were observed only upstream the *hmp* genes. Multiple experimental studies in *B. subtilis* showed nitrite- or NO-dependent induction of expression of *hmp*; however, the mechanism of this control was not known [9,29]. The experimentally mapped *hmp* promoter in *B. subtilis* significantly overlaps with the predicted tandem NsrR sites [44].

**Figure 7:**
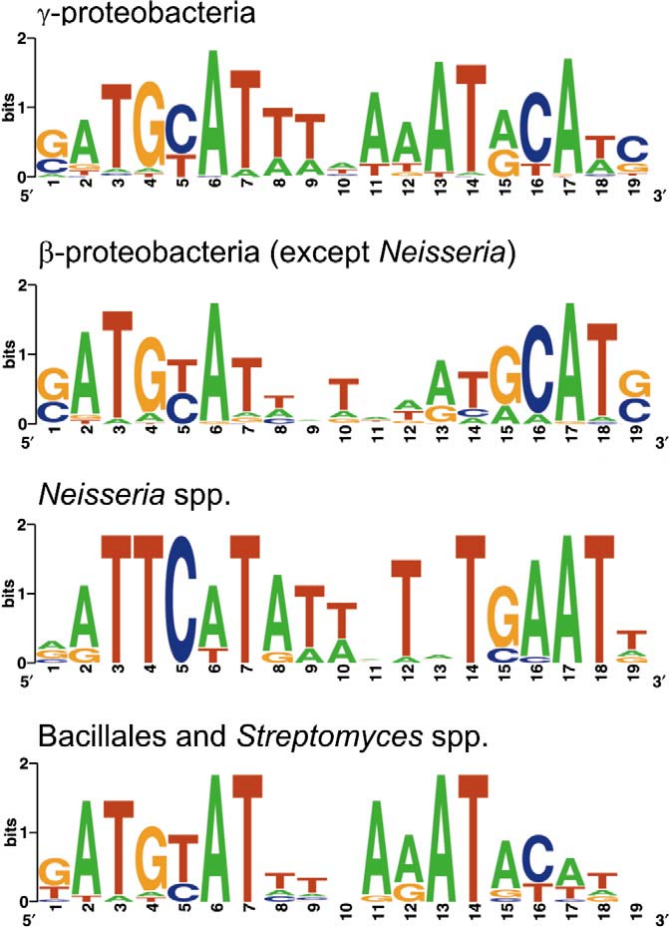
Sequence Logos for the Identified NsrR-Binding Sites in Various Bacterial Taxa

The obtained data suggest that the nitrite-responsive NsrR regulon has a wide phylogenetic distribution. Its most conserved member is the NO-detoxifying flavohemoglobin Hmp, which is present both in Gram-negative and Gram-positive bacteria. Most other regulon members are involved in the nitrosative stress and denitrification. The identified NsrR recognition motif, a palindrome with consensus gATGyAT-(N_5_)-ATrCATc, is well conserved in most analyzed bacteria (Figure 7). The only exception is the NsrR recognition motif in *Neisseria* species, where symmetrical positions G_4_ and C_16_ are replaced by T and A, respectively.

### NorR Regulon

In Vibrionales, the *hcp-hcr* operon is preceded by a gene that encodes a homolog of the NO-responsive regulator NorR, named NorR2. NorR is a σ^54^-dependent transcriptional activator that regulates expression of the NO reductase operons, *norVW* in *E. coli* and *norAB* in *R. eutropha* [21,22]. NorR binds to three tandem operator regions, inverted repeats with degenerate consensus GT-(N_7_)-AC, which are localized upstream of the σ^54^ promoter site [23]. By applying the motif detection procedure to the *hcp* promoter regions from four Vibrionales genomes, we identified two tandem palindromic sites with consensus GATGT-(N_7_)-ACATC (Figure S4). These likely binding sites for the NorR2 protein are localized immediately upstream of candidate σ^54^ promoters well conforming to the consensus, and thus could be involved in the NO-dependent activation of the *hcp-hcr* operon (Table S5). In addition, the *norR2-hcp-hcr* gene loci in Vibrionales contain a single NorR site without an associated σ^54^ promoter located upstream of the *norR2* gene. This site could be involved in the negative autoregulation of the NorR2 expression (Figure 6).

To analyze analogous NO-responsive regulons in other species, we performed exhaustive similarity search and identified *norR*-like genes in only a limited number of β- and γ-proteobacteria (Figures 4 and 6). An *E. coli*-like arrangement of the divergently transcribed *norR* and *norVW* genes with conserved tandem NorR-binding sites and a σ^54^ promoter was found in *S. typhimurium,* two *Erwinia* species, and two *Vibrio* species. Other NO-detoxification genes possibly regulated by candidate NorR sites are the NO-reductase *norB* in *Ralstonia* spp. and *Shewanella putrefaciens,* and the NO dioxygenase *hmp* gene in *V. cholerae, Pseudomonas* spp., *Polaromonas* sp., and *B. fungorum*. In all these cases except *P. stutzeri,* the *norR* gene is clustered with the target genes on the chromosome. In the unfinished genome of *P. stutzeri,* the candidate tandem NorR sites followed by candidate σ^54^ promoters were found upstream of the *hmp* and *dnrN* genes, but the *norR* gene was not found in the sequenced portion of the genome. In addition, we found that *V. cholerae* has a second target for NorR in the genome, the hypothetical gene *nnrS,* which was identified as a member of various NO/nitrite-responsive regulons in other proteobacteria (NsrR, DNR, NnrR, see below).

**Figure 8:**
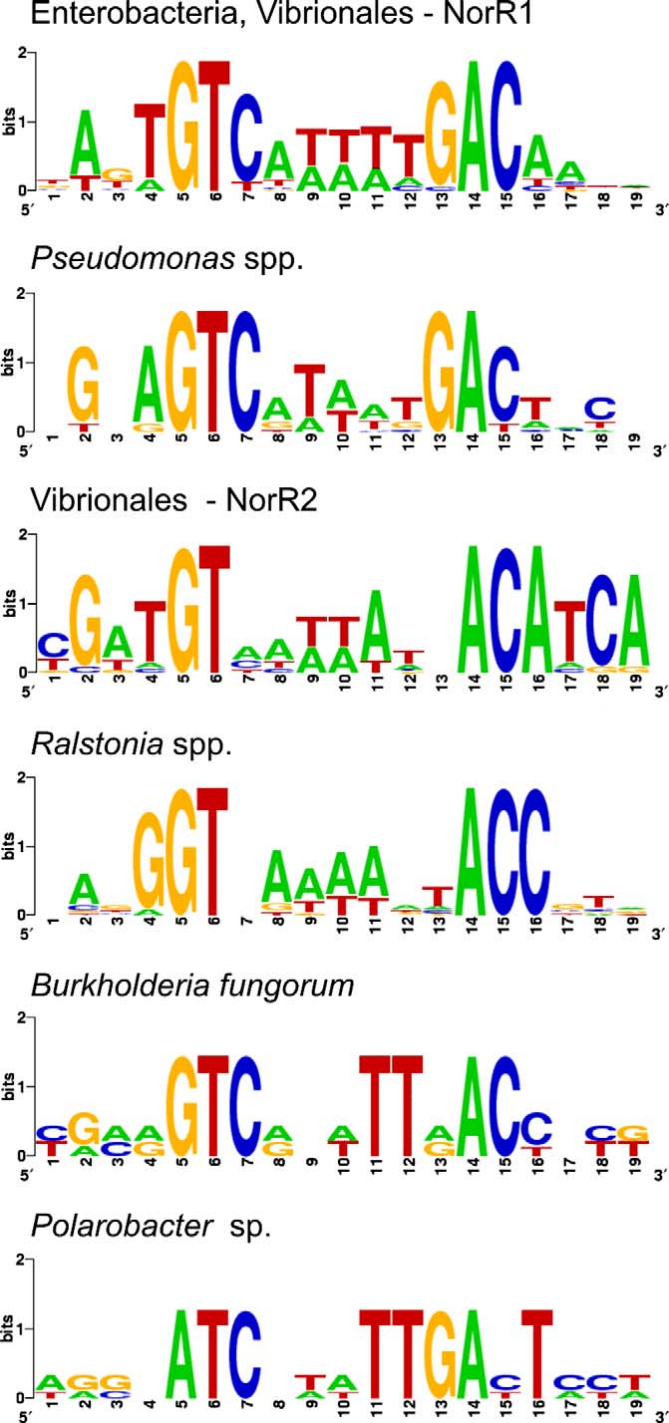
Sequence Logos for the Identified NorR-Binding Sites in Various Species of Proteobacteria

The consensus sequences of NorR and NorR2 recognition motifs identified in various taxonomic groups have only a limited number of universally conserved positions (Figure 8). Positions G_5_ and T_6_ and complementary positions A_14_ and C_15_ are the most conserved ones throughout the NorR family, being only partially replaced in *Polaromonas* sp. (A_5_). Noteworthy, in some *Vibrio* species, two *norR* paralogs are present, *norR1* and *norR2,* which are associated with the *norVW* and *hcp-hcr* operons, respectively. The NorR1 and NorR2 consensus sequences differ significantly in four positions (C_7_, G_13_ for NorR1 and G_2_, C_18_ for NorR2), allowing for discrimination of the target sites by the NorR paralogs in these species.

### Complex Regulation of Hybrid Cluster Protein Genes

**Figure 9:**
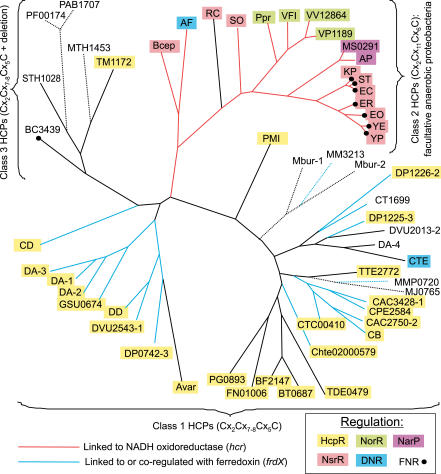
Maximum Likelihood Phylogenetic Tree of the Hybrid Cluster (Prismane) Proteins Genes predicted to be regulated by the nitrogen oxides–related factors are highlighted by respective colors. Additionally, predicted FNR-regulated genes are denoted by black circles. Genes positionally linked to the NADH oxidoreductase *hcr* and the hypothetical ferredoxin *frdX* genes are shown by red and blue lines, respectively. Archaeal genes are shown by pointed lines.

Differences in the predicted mode of regulation of the hybrid cluster proteins (Table 1), which are present in diverse bacterial and archaeal species, are well traced on the phylogenetic tree of this protein family (Figure 9). Indeed, the *hcp* gene is regulated by HcpR (highlighted in yellow) in many anaerobic bacteria, by NsrR in facultative anaerobic enterobacteria, and some β- and α-proteobacteria (in magenta), by NorR in most Vibrionales (in green), and by DNR in *A. ferrooxidans* and *Thermochromatium tepidum* (in blue). It often forms operons with either the NADH oxidoreductase *hcr* (in γ-proteobacteria) or the ferredoxin-like gene *frdX* (in δ-proteobacteria and *Clostridium* spp.), suggesting functional linkage between the Hcp and Hcr/FrdX proteins. In addition to predicted NsrR sites, the *hcp-hcr* operons in enterobacteria are also preceded by candidate binding sites of the anaerobic activator FNR, suggesting their induction during anaerobiosis (Figure S1). We also investigated the regulatory regions of *hcp* in two Pasteurellaceae lacking all above-mentioned nitrogen oxides regulators *(Actinobacillus pleuropneumoniae* and *Mannheimia succiniciproducens)* and found a strong candidate binding site of NarP, a response regulator from the nitrate/nitrite-specific two-component regulatory system NarQ-NarP. The NarP regulon in *E. coli* contains mainly genes from the nitrate/nitrite respiration pathway [18], whereas in the above two Pasteurellaceae, the NarP regulon is extended to include the detoxification genes *hcp-hcr, dnrN,* and *norB*. Additional analysis using the NarP recognition rule revealed candidate NarP sites upstream of some NsrR-regulated genes in γ-proteobacteria and *Neisseria* species (Figure 6). In agreement with these findings, the nitrate- and nitrite-induced transcription from the *hcp* promoter in *E. coli* was found to depend on the response regulator proteins NarL and NarP [45]. These observations show that the nitrite induction of the NO-detoxification genes in different genomes can be achieved by multiple transcriptional factors.

**Table 1:**
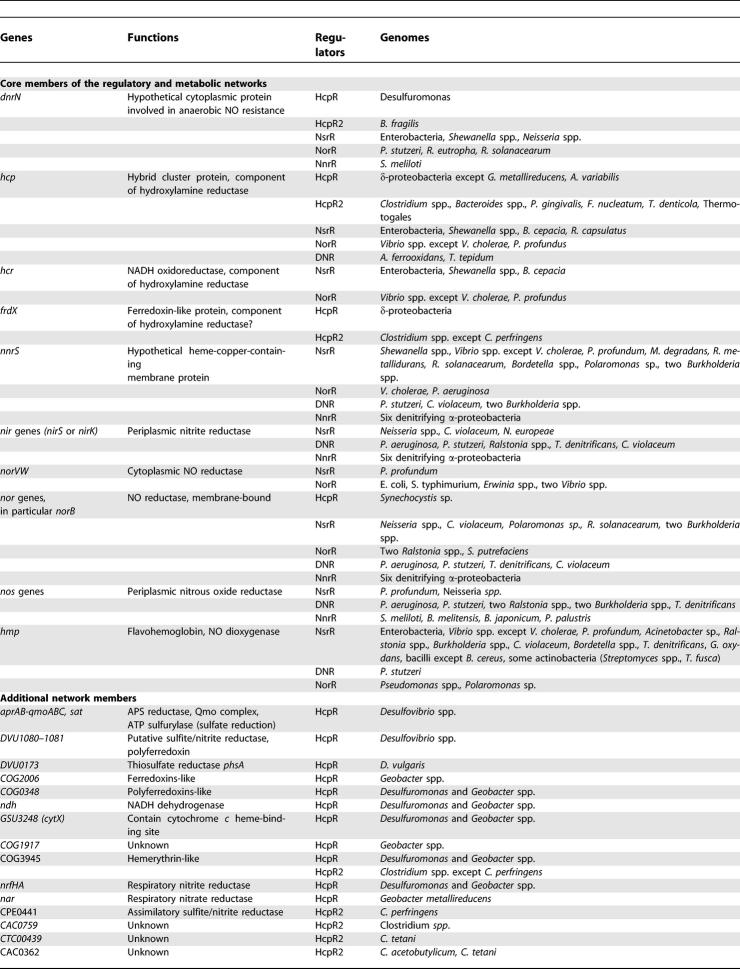
Predicted Members of Regulatory and Metabolic Networks of the Nitrogen Oxides Dissimilatory Metabolism

**Table 1:**
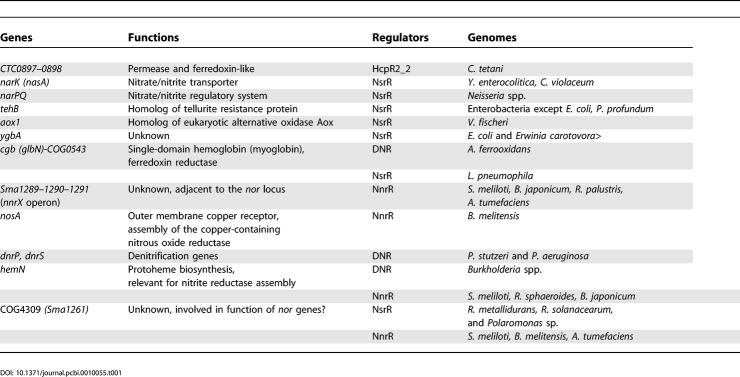
Continued

### Additional Members of the Regulons

**Figure 10:**
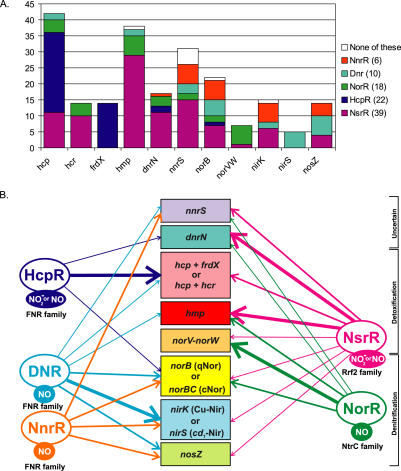
Regulatory Interactions between Genes for Dissimilatory Nitrogen Oxides Metabolism in Bacteria (A) Distribution of regulators and regulated genes. The number of cases when a gene is regulated by a specific transcription factor is indicated by the length of a colored bar in the histogram. The white bar in the histogram shows the cases when the gene is present in a genome possessing at least one of the studied regulons, but is not regulated by any of them. (B) Combined regulatory network. Arrows denote regulatory interactions, with the thickness reflecting the frequency of the interaction in the analyzed genomes. Experimentally established (for DNR, NnrR, NsrR, and NorR) and predicted based on the regulon content (for HcpR) signal molecules are shown in filled ovals, and the protein family for each transcription factor is shown below.

The main regulatory interactions analyzed in this study are shown in Figure 10 and Table 1. The core regulon members, that is, genes regulated by the nitrogen-oxide responsive factors NsrR, HcpR, DNR, NnrR, and NorR in many genomes, are the hybrid cluster protein gene *hcp,* the NO-detoxifying flavohemoglobin *hmp,* two hypothetical genes *dnrN* and *nnrS,* NO reductase operon *norVW,* and multiple denitrification genes, *nir, nor,* and *nos,* encoding nitrite, NO, and nitrous oxide reductases, respectively. The core regulon members can be regulated by different regulators in various genomes. Further, some genes may be regulated by several regulators simultaneously. All considered regulons also contain a large number of additional members, which are summarized in Table 1.

Though the main target of the HcpR regulators are genes encoding the hybrid cluster protein Hcp and the hypothetical ferredoxin-containing protein FrdX, the HcpR regulons are significantly extended in δ-proteobacteria and clostridia (Table S1, Figure 2, and Table 1). In *Desulfuromonas* and *Geobacter* species, they include the nitrite reductase *nrfHA,* the NADH dehydrogenase *ndh,* and the nitrate reductase *nar* operon. In *Desulfovibrio* species, the HcpR regulon is extended to include the *apsBA* and *sat* loci involved in the sulfate reduction pathway. Among hypothetical genes, the predicted HcpR regulons often contain ferredoxin-, hemerythrin-, or cytochrome *c*-like genes. For instance, the *CTC0897-CTC0898* operon of *C. tetani* encoding a permease and a ferredoxin-like protein, respectively, is likely regulated by the divergently transcribed paralog of HcpR2 (CTC0896).

Additional members of the NsrR regulon were identified in all enterobacteria (Figure 6). Two hypothetical transporter genes, that are homologous to the tellurite resistance gene *tehB* and the nitrite extrusion gene *narK,* could be involved in the protection from the nitrosative stress by excretion of nitrite from cytoplasm. In support of these observations, the NsrR regulons were found to include a *narK*-like transporter gene *(nasA)* in β-proteobacterium *C. violaceum* and a *tehB* homolog in *Photobacterium profundum*. The *V. fischeri* NsrR regulon includes a homolog of the eukaryotic alternative oxidase Aox. In *Legionella pneumophila,* a non-denitrifying γ-proteobacterium without *hmp, hcp, dnrN,* and *nnrS* genes, the *glbN-lpg2536* operon encoding a heme-containing cyanoglobin and a hypothetical ferredoxin reductase was found to be a sole NsrR target. We suggest that both these genes could be involved in the detoxyfication process by mediating NO oxidation (similar to the flavohemoglobin Hmp).

Denitrifying bacteria like the *Pseudomonas* and *Burkholderia* species contain additional members of the DNR and NnrR regulons (Figures 4 and 5). For instance, the *hemN* gene encoding an O_2_-independent coproporphyrinogen III oxidase involved in the protoheme biosynthesis and relevant for denitrification [46] is preceded by a strong DNR site in the *Burkholderia* species but has candidate NnrR-binding sites in some α-proteobacteria. In *Brucella melitensis,* the NnrR regulon includes the *nosA* gene, encoding an outer membrane copper receptor, shown to be required for the assembly of the copper-containing nitrous oxide reductase in *P. stutzeri* [47]. Finally, a gene of unknown function *(COG4309)* is probably co-transcribed with the NnrR-regulated *nor* genes in three rhizobial species, where as in three β-proteobacteria this gene is a predicted member of the NsrR regulon and it is often co-transcribed with *norB* (Figure 4). These observations indicate that the COG4309 family could be relevant for function of the NO reductase.

Two close *dnr* paralogs, most likely resulting from a recent duplication, were found in *A. ferrooxidans*. One is divergently transcribed with the *hcp-COG0543* operon, which is preceded by a strong candidate DNR site. The second paralog clusters with the *cgb-COG0543-COG0446* operon, which also is preceded by a candidate DNR site. The product of the first gene in this operon is similar to a single-domain hemoglobin in *Campylobacter jejuni,* whish is indispensable for defense against NO and nitrosating agents [48]. Thus we predict that the recently duplicated DNR paralogs in *A. ferrooxidans* regulate two different NO-detoxifying systems.

## Discussion

The results of this study demonstrate considerable variability of the metabolic and regulatory systems for nitrogen oxides (Figure 10). Many genes change the regulators in different genomes (Table 1). However, overall, the system seems to be quite conserved. Genes involved in denitrification, such as *nir, nor,* and *nos,* are mainly regulated by two NO-responsive transcriptional activators from the FNR/CRP family, NnrR in α-proteobacteria and DNR in β-proteobacteria, and *Pseudomonas* spp. Three different nitrogen oxides-responsive transcription factors appear to regulate genes required for defense against the nitrosative stress: the σ^54^-dependent activator NorR from the NtrC family in some γ- and β-proteobacteria (present in at least 18 species), the Rrf2 family NsrR repressor widely distributed in proteobacteria and firmicutes (39 species), and the FNR-like transcription factor HcpR in diverse anaerobic bacteria (22 species). The primary targets of the newly identified regulator HcpR are the hybrid-cluster protein Hcp, which has a protective role in nitrite stress conditions, and the associated ferredoxin-like proteins. NorR usually regulates cytoplasmic NO reductase *norVW* and sometimes another membrane-bound NO reductase *(norB)* and the NO dioxygenase *hmp*. The NsrR regulon almost always includes the *hmp* and *hcp* genes, as well as one or both genes of unknown function, *dnrN* and *nnrS*.

On the whole, the NsrR, NorR, and DNR regulons are differentially distributed in γ- and β-proteobacteria, and the former prevails over the other two. All three regulons are present only in three *Ralstonia* species, where NsrR controls the NO-detoxifying flavohemoglobin, whereas NorR and DNR regulate the denitrification genes *(nir, nor,* and *nos).* In addition, the NorR and NsrR factors co-occur in ten other non-denitrifying species, complementing each other in the control of the nitrosative stress genes. Finally, NorR and DNR regulators were found only in *P. aeruginosa,* and NsrR and DNR co-occur in four denitrifying β-proteobacteria.

Some regulatory interactions in the identified core regulatory network seem to be taxon-specific (thin lines in Figure 10B; see also “Complex regulation of hybrid cluster protein genes” above). They include NsrR-regulated *norVW* and *nos* in *P. profundum, norB* and *nirK* in various β-proteobacteria; NorR-regulated *dnrN* in *P. stutzeri* and *nnrS* in *P. aeruginosa*; HcpR-regulated *dnrN* in *B. fragilis* and *Desulfuromonas*, *norB* in *Synechocystis* sp.; and DNR-regulated *nnrS* and *hmp* in *P. stutzeri, hcp* in *A. ferrooxidans* and *T. tepidum*. The extensions of the NsrR regulon include the denitrification genes *nirK* and *norB* in *Neisseria* species, *Burkholderia* spp., and *C. violaceum*. The former is of particular interest as, in contrast to the latter two lineages, the *Neisseria* species lack the DNR regulator, assuming a lineage-specific substitution of both the transcription factor and its binding sites. Indeed, in the *Neisseria* species, the complete denitrification pathway including nitrite, NO, and nitrous oxide reductases, as well as *dnrN* and the two-component regulatory system *narQP,* seems to be regulated by NsrR. The hypothesis that NsrR mediates regulation of denitrification genes in *Neisseria* is further supported by the observation that in *N. gonorrhoeae,* the *norB* gene is strongly induced by NO independently of FNR and NarP [30].

Not only genes are shuffled between regulons in different genomes, but there may exist considerable interaction between regulators. Firstly, in some species the DNR regulon overlaps with other nitrogen oxide-responsive regulons. The upstream regions of *norB* and *nirK* in *C. violaceum, COG4309-norB* in *R. solanacearum,* and *nnrS2* in *Burkholderia* species contain two candidate regulatory sites, a downstream NsrR site and an upstream DNR site (Figure 4), yielding both positive regulation by the NO-responsive activator DNR and nitrite-induced de-repression by the NsrR repressor. Secondly, the NO-detoxifying gene *hmp* in *P. stutzeri* is preceded by three candidate NorR sites at positions −192, −173, and −148 (relative to the translational start site), a strong DNR site at position −116, and a putative σ^54^ promoter at position −91 (Figure 6). This arrangement of regulatory elements indicates dual positive control of the *hmp* expression by different NO-responsive activators, σ^54^-dependent NorR and σ^70^-dependent DNR. Finally, in many cases genes are regulated by two additional regulators, the oxygen-responsive factor FNR (*hcp-hcr, hmp,* and *narK* in enterobacteria) and the nitrite/nitrate-sensitive two-component system NarQ/NarP (*hcp-hcr, dnrN,* and *hmp* in enterobacteria, *nnrS* in Vibrionales and *Shewanella* spp.). More complex regulatory interactions are observed in *Neisseria* spp., where NsrR regulates the NarQ/NarP system, whereas the common upstream region of the divergently transcribed genes *norB* and *nirK* contains two candidate NsrR sites, a candidate NarP site in the middle, and an FNR-binding site immediately upstream of *nirK,* the latter being involved in the anaerobic induction of this gene [49].

Various aspects of the described regulatory network for the nitrogen oxides metabolism are verified by a large number of independent experimental observations. Upregulation of the *hcp* gene in response to growth on nitrate or nitrite was reported in *S. oneidensis, E. coli, S. typhimuruim,* and *D. vulgaris* [10–12,14]; the same pattern of regulation was observed for *dnrN* in *S. typhimuruim* and *P. stutzeri* [12,34]. Our prediction of positive regulation of *hcp-frdX* and negative regulation of the sulfate reduction genes *apsBA* and *sat* [33] was validated in a macroarray hybridization study, where *hcp* was upregulated 255-fold with 5 mM nitrite, whereas *aprAB* and *sat* were downregulated nearly 10-fold in the same conditions [14]. In addition, nitrite induced transcription of thiosufate reductase *phsA* (a candidate member of the HcpR regulon) and inhibited the membrane-bound electron transport complex *qmoABC,* located just downstream of the *apsBA* genes and thus also possibly repressed by HcpR.

The flavohemoglobin gene *hmp* is induced by NO and nitrite in *E. coli* and *B. subtilis* [28,29], and the mechanism of the *hmp* regulation by the nitrite repressor NsrR predicted in this study is conserved in these diverse bacteria. Another NO-mediated mechanism of *hmp* regulation in *E. coli* by the O_2_-responsive repressor FNR was proposed [50]. At that, NO was found to inactivate FNR anaerobically, restoring the *hmp* expression. However, our data indicate that, in contrast to the candidate NsrR site, the FNR binding site is conserved in only closely related bacteria *E. coli* and *S. typhimurium* (Figure S3). Finally, NO induces the *norB* expression in *N. gonorrhoeae,* but it was found to be independent of known nitrogen oxide-responsive regulators [30]. Here we describe possible co-regulation of all denitrification genes in the *Neisseria* species by the nitrite-sensitive repressor NsrR. Recently, transcriptional regulation of the flavohemoglobin gene *fhp* (*hmp* ortholog) by the NO-responsive regulator FhpR (NorR ortholog) has been demonstrated in *P. aeruginosa* [51].

A recent paper by Elvers et al. [52] describes a new nitrosative stress-responsive regulon in ɛ-proteobacterium *C. jejuni* regulated by a member of the CRP/FNR family. This regulator (named NssR) is homologous (26% identity) to the HcpR2 factor from *T. maritima* described in this study. It has an FNR-like recognition motif (TTAACnnnnGTTAA) and specificity-determining positions A_180_ and Q_181_, conforming to the correlation between these two positions and contacting bases in the DNA motif observed in this study. NssR positively regulates expression of the *Campylobacter* globin gene *cgb,* encoding a single-domain hemoglobin that mediates resistance to NO and nitrosative stress [48]. Thus the regulation of the *cgb* gene by HcpR in *A. ferrooxidans* predicted in this study is in agreement with verified NssR-mediated activation of the *cgb* ortholog in *C. jejuni*.

While this study was being completed, we obtained a personal communication from S. Spiro from Georgia Institute of Technology, Atlanta, Georgia, United States, that the NsrR ortholog in *E. coli* (YjeB) is a NO-sensitive transcriptional repressor of several nitrosative stress responsive genes including *hmp, ytfE (dnrN),* and *ygbA*. Moreover, a common inverted repeat sequence coincided with the defined here NsrR recognition motif was shown to be involved in NsrR-mediated repression of the *ytfE* gene.

The mechanisms of regulation of nitrogen oxides metabolism in bacteria are of great importance both for ecology and medicine. Nitrifying and denitrifying microorganisms are significant sources of both nitric and nitrous oxides production in the atmosphere, and thus have a great impact in the greenhouse effect [53]. Nitrate has become a pollutant of groundwater and surface water. NO and reactive nitrogen intermediates are also part of the arsenal of antimicrobial agents produced by macrophages [54]. Therefore, nitrosative stress tolerance genes, which are inducible after invasion, provide a strong advantage for pathogenic bacteria that need to resist the host defense system. Here we tentatively characterized the transcriptional regulatory network for the genes involved in these significant metabolic processes. Overall, although each particular prediction made in this study may require experimental verification, the emerging overall picture seems to be rather consistent and robust.

## Materials and Methods

Complete and partial bacterial genomes were downloaded from GenBank [55]. Preliminary sequence data were also obtained from the Institute for Genomic Research (http://www.tigr.org), the Wellcome Trust Sanger Institute (http://www.sanger.ac.uk/), and the DOE Joint Genome Institute (http://jgi.doe.gov). The gene identifiers from GenBank are used throughout. Genome abbreviations are listed in Table 2. Protein similarity search was done using the Smith-Waterman algorithm implemented in the GenomeExplorer program [56]. Orthologous proteins were defined by the best bidirectional hits criterion and named by either a common name of characterized protein or by an identifier in the Clusters of Orthologous Groups (COG) database for uncharacterized proteins [57]. Distant homologs were identified using PSI-BLAST [58]. The SEED tool, which combines protein similarity search, positional gene clustering, and phylogenetic profiling of genes, was applied for comparative analysis and annotation of multiple microbial genomes (see the “Nitrosative stress and Denitrification” subsystems at http://theseed.uchicago.edu/FIG/index.cgi). The phylogenetic trees were constructed by the maximum likelihood method implemented in PHYLIP [59] using multiple sequence alignments of protein sequences produced by CLUSTALX [60]. In addition, the InterPro [61], and PFAM [62] databases were used to verify protein functional and structural annotations.

**Table 2:**
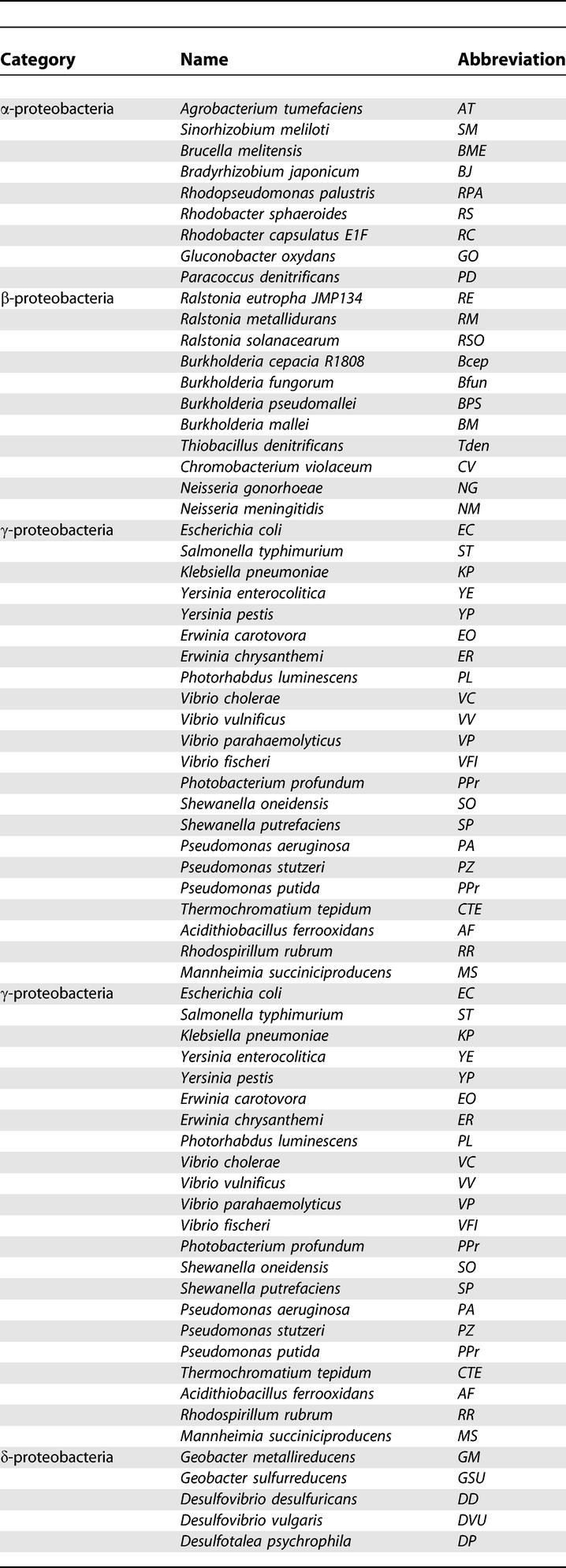
List of Genome Abbreviations Used in This Study

**Table 2:**
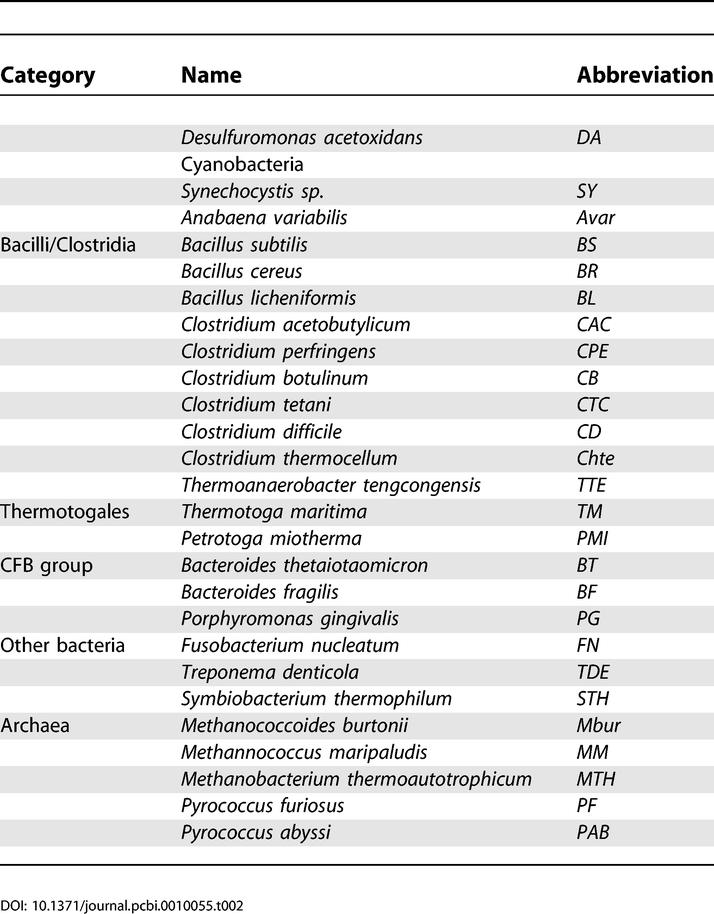
Continued

For identification of candidate regulatory motifs, we started from sets of potentially co-regulated genes (using previous experimental and general functional considerations). A simple iterative procedure implemented in the program SignalX (as described previously in [63]) was used for construction of common transcription factor–binding motifs in sets of upstream gene fragments. Each genome encoding the studied transcription factor was scanned with the constructed profile using the GenomeExplorer software (see the detailed description at http://bioinform.genetika.ru/projects/reconstruction/index.htm), and genes with candidate regulatory sites in the upstream regions were selected. The threshold for the site search was defined as the lowest score observed in the training set. Dependent on the DNA motif and the number of sites in the training set, such threshold could be too strict or too permissive. It seems that the threshold choice was adequate in our cases, as little clear false positives were encountered, and, on the other hand, most functionally relevant genes were found to belong to at least one of the studied regulons (Table 3 and Results and Discussion sections). The upstream regions of genes that are orthologous to genes containing regulatory sites of any of studied nitrogen-related factors were examined for candidate sites even if these were not detected automatically with a given threshold. Among new candidate members of a regulon, only genes having candidate sites conserved in at least two other genomes were retained for further analysis. We also included new candidate regulon members that are functionally related to the nitrogen oxides metabolism. This procedure allowed us to reject a small number of false positive sites identified after scanning of microbial genomes (Table 3). Sequence logos for derived regulatory motifs were drawn using WebLogo package v.2.6 [64] (http://weblogo.berkeley.edu/).

**Table 3:**
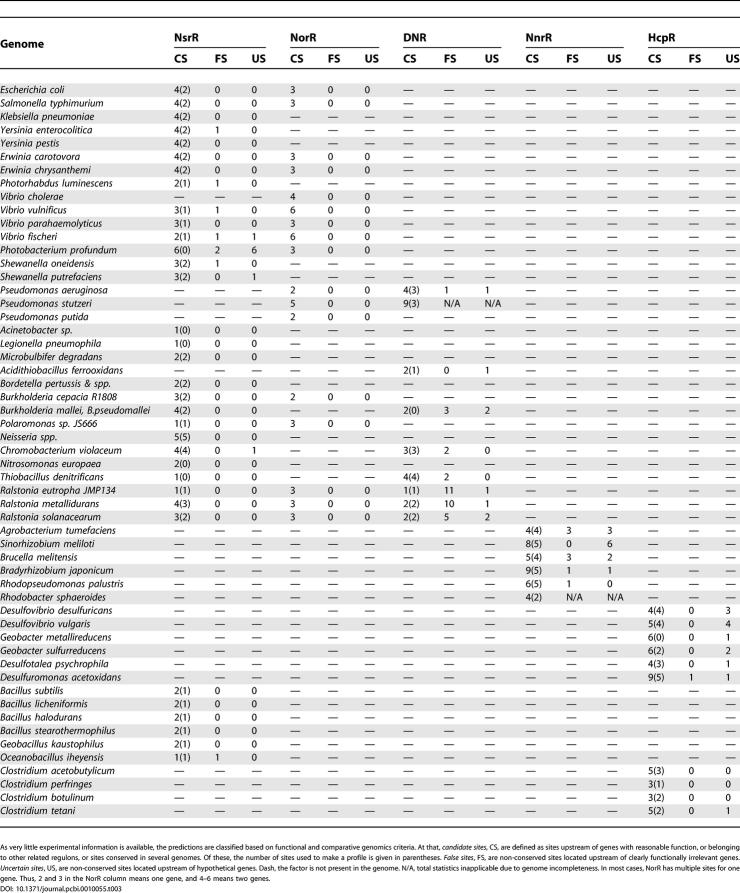
Distribution of Predicted Regulatory Sites in Bacterial Genomes

## Supporting Information

Figure S1 Multiple Sequence Alignment of the Upstream Regions of the hcp-hcr Operons from Enterobacteria (22 KB DOC)

Figure S2 Multiple Sequence Alignment of the Upstream Regions of the dnrN Genes from Enterobacteria (20 KB DOC)

Figure S3 Multiple Sequence Alignment of the Upstream Regions of the hmp Genes from Enterobacteria (26 KB DOC)

Figure S4 Multiple Sequence Alignment of the Upstream Regions of the hcp-hcr Operons from Vibrio Species (24 KB DOC)

Table S1 Candidate HcpR-Binding Sites (40 KB XLS)

Table S2 Candidate DNR-Binding Sites (34 KB XLS)

Table S3 Candidate NnrR-Binding Sites (29 KB XLS)

Table S4 Candidate NsrR-Binding Sites (24 KB XLS)

Table S5 Candidate NorR-Binding Sites and Corresponding σ^54^ Promoters (23 KB XLS)

### Accession Numbers

The Pfam (http://www.sanger.ac.uk/Software/Pfam/) accession numbers for products discussed in this paper are: hypothetical transcriptional factor from Rrf2 protein family (PF02082) and eukaryotic alternative oxidase Aox (PF01786).

Complete and partial bacterial genomes were downloaded from GenBank [55].

## Abbreviation

NO: nitric oxide.
