# Supplementary material for: Dissimilatory Metabolism of Nitrogen Oxides in Bacteria: Comparative Reconstruction of Transcriptional Networks
Source: PLoS Comput Biol. 2005 Oct 28;1(5):e55. doi: 10.1371/journal.pcbi.0010055 (PMC1274295; doi:10.1371/journal.pcbi.0010055)
Supplement: Figure S1 — (22 KB DOC) [file pcbi.0010055.sg001.doc]

**Supplementary Figure S1.** Multiple sequence alignment of the upstream regions of the *hcp-hcr* operons from enterobacteria. Genome abbreviations are listed in Table 2. Candidate binding sites for transcription factors NsrR and FNR are highlighted in green and yellow, respectively. Candidate NarP-binding sites are shown in red. The experimentally mapped transcription start site is in bold and underlined. The *hcp* start codons are in bold.

ST_hcp ---------------------------------GGCCTGATACCTCTTTGGCGGTAG-GC

KP_hcp TTTGCTGGTCCCGATCCTGATCGCCTTTTTTACCGCCTGAGCACCCGCTGGCGGCAG-CC

EC_hcp -------------------------------------TAATACCTCTCTGGCGGTAG-AT

ER_hcp GCCCTACGCCTCGGATCTGCCTCGCGACGCATAAAAATGCCCTGTGAATGCGAGTGA-TT

EO_hcp TGCACATTCATCGTCGTTTTCTCCCT-CGTTTA----TGGGCTGTGATTGAGAGTGA-TT

YP_hcp ATCTCCCAGTCGCCCCCCCCCGCCCTCTGGGGGCTTTTCCTTCCCTATATCGAATAAATT

YE_hcp TTCGCGCAGT-GAGCCACTTGGTTCACTGCGTAATTTCACT--CCAATAGTGAATAAATT

ST_hcp ATC-T-----GCCGCCAAAATTGCGCTAAATCAATCTCCCTGCACATTATGGCAGAAATC

KP_hcp CTCCT-----GCCGCCAAATTTGCGCTAAATCAATCTCCCTTTAAGTTGTCTTAGAAATA

EC_hcp CCC-T-----GCCGCCAAAATTGCGCTAAATCAATCTCCCTTAAAGTTGCATGAAAAATC

ER_hcp GTCAT---CCACAAGTAAAATTGCGCTAAATCAACTTCGCCTTAAATTGCCTATAAAAGA

EO_hcp TTCATTGCCCATCACCAAACTTGCGCTAAATCAAATTTACCTAAAGTTGCATTAAAAAAG

YP_hcp CTCAT---TCGCAGTAATAATTGCGTCAAATCAATATAAGTGAATATTGCATTAAAAATA

YE_hcp CTCAT---TCGCAGCAAAAGTTGCGCCAAATCAAAACAAGTTAAAGTTGCATTAAAAAAG

* * * * ***** ******* * ** ***

ST_hcp CCTTTTATCCCTTTGTTCACAGGCATAACCTT--AAACATGTATATTAAATATAACTTTA

KP_hcp CCTTTTCACCCGTTGCCGCGCGGCATAACCTT--AAACATGTATATCAAATATAACTTTA

EC_hcp CCTTTTATCCCCGCGTTAAGCGTCTTAACCTT--AAACATG**T**ATATTAAATATAACTTTA

ER_hcp TCTTTTTAGACATCGGCAACCGTTCTATGCTTTTAAACAAGCATTATAAATACAACTTTA

EO_hcp CCTTTTTACCTGCCTCGGCTGACACTATGCTTT-AAACAAGCATTTAAAATGCAACTTAA

YP_hcp CCTTTTTAAACCGATGGCGTATCTCTATGCTCCAAAACACGCATTACAAATACAACTTTA

YE_hcp CCTTTTTAAACCGCTTGTGCACATCTATGCTTTTAAACACGCATTACAAATGCAACTTTA

***** ** ** ***** * ** **** ***** *

ST_hcp AAAGGTGTGACC---**ATG**TTTTGTGTGCAATGTGAACAAACCATCCGTACACCAGCCGGA

KP_hcp AAGGTGTGACC----**ATG**TTTTGTGTGCAATGTGAACAAACCATTCGTACCCCAGCCGGC

EC_hcp AAAGGTGTGATC---**ATG**TTTTGTGTGCAATGTGAACAAACTATCCGTACTCCGGCAGGA

ER_hcp AAAAAGGAAGCCATT**ATG**TATTGTGTGCAATGTGAACAGACCATCCGGACCCCGGCCAGT

EO_hcp AAAAAGGAAGTCATC**ATG**TTTTGTGTGCAATGTGAACAAACGATTCGTACCCCTGTTGGA

YP_hcp AAAAGGAAAATAATA**ATG**TTCTGTGTGCAATGTGAACAAACTATCCGAACGCCAGCAGGT

YE_hcp AAAGGAAAAATAATT**ATG**TTCTGTGTGCAATGTGAACAAACTATCCGGACGCCGGTGGGT

** **** ***************** ** ** ** ** ** * *
