# Supplementary material for: Dissimilatory Metabolism of Nitrogen Oxides in Bacteria: Comparative Reconstruction of Transcriptional Networks
Source: PLoS Comput Biol. 2005 Oct 28;1(5):e55. doi: 10.1371/journal.pcbi.0010055 (PMC1274295; doi:10.1371/journal.pcbi.0010055)
Supplement: Figure S2 — (20 KB DOC) [file pcbi.0010055.sg002.doc]

**Supplementary Figure S2.** Multiple sequence alignment of the upstream regions of the *dnrN* genes from enterobacteria. Genome abbreviations are listed in Table 2. Candidate NsrR binding sites are highlighted in green. Candidate NarP-binding sites are shown in red. The *dnrN* start codons are in bold.

EC_ytfE ----------------------CTCAGAAAGTTGACAC-------GCTGGCAG-TGAGTT

KP_dnrN GGTCTCCGCCGTACGCGCCAGACCGGAAAAACCGATGCTGCAGCCGCTGGCGGACGAAAA

ST_dnrN -----------------CCGGTCACGGAACGCTAATCTCAT----CCTGGC-------TT

ER_dnrN ----------CAGTAACCCCATCCAACCGCGCTTCCACGGC----GCGGTTTTTTTCGCC

EO_dnrN ------------------------------------ACCGTG--CAGTAATACGGAATGC

YP_dnrN --TCTCTGGCGGCAACGCCTTACACTGTACGCTGCTGTCACC--CCATAATACCCACTTG

EC_ytfE AAATAAGCCTCTGCTACGTAAGGGTTATAGCTTTTGCCT--TAAAGATGCATTTAAAATA

KP_dnrN AAAGTAGCCTCGCCTCCTTAAGGGGTATT-CCCTCGCCA--AAAAGATGCATTTAAAATA

ST_dnrN AAATTATACG-CACCCCGAAAGAGTTATA-GCCTCGCCT--TAAAGATGCATTTAAAATA

ER_dnrN GTCTTCCCCGGTAATCCTTTTTAGGGATA-GCTAATCTT--AAAAGATGCATTTAAAATA

EO_dnrN ATTTTACCGCCTACCTCGTAAGCGGTATA-TTCAACGCT--AAAAGATGCATTTAAAATG

YP_dnrN GAGTTATATCACAATCTATAACATGCATTTATTATGCATGTTATAGGTGTATAATAAATA

** * ** ** ** ****

EC_ytfE CATCTTATCT------TATTAAG-AATGAGGTATCAGCT**ATG**GCTTATCGCGACCAACCT

KP_dnrN CATCTTATAT------TCCTGAT-GACGAGGTAACTGCT**ATG**GCTTTCCGTGACCAACCT

ST_dnrN CAACTTATAT------TATTGCA-AATGAGGTAACGGCT**ATG**GCTTATCGCGATCAACCT

ER_dnrN CATTTTATAAATTA--CACCGTT-CAGGGGGTACCCACC**ATG**CATTACCGCGACCAATCA

EO_dnrN CAATTTATAAACTG--CATTTAT-TGAGAGGCATC-ATC**ATG**GCATACCGCGATCAATCC

YP_dnrN CATGTTATAGATATATAATTACTCAATGAGGTGTC-ATT**ATG**GATTACCGCAATCAGTCT

** **** * ** * *** * ** * ** *
