# Supplementary material for: Dissimilatory Metabolism of Nitrogen Oxides in Bacteria: Comparative Reconstruction of Transcriptional Networks
Source: PLoS Comput Biol. 2005 Oct 28;1(5):e55. doi: 10.1371/journal.pcbi.0010055 (PMC1274295; doi:10.1371/journal.pcbi.0010055)
Supplement: Figure S3 — (26 KB DOC) [file pcbi.0010055.sg003.doc]

**Supplementary Figure S3.** Multiple sequence alignment of the upstream regions of the *hmp* genes from enterobacteria. Genome abbreviations are listed in Table 2. Candidate NsrR binding sites are highlighted in yellow. Candidate NarP and FNR binding sites are shown in red and blue, respectively. The experimentally mapped transcription start site is in bold and underlined. The *hmp* start codons are in bold.

[ST_hmp](http://ergo.integratedgenomics.com/ERGO/CGI/prot.cgi?prot=RTY05232&user=rodionov:40164470) AACGA-GAGCCTGACGAATTTCAACGGATTTCTTTTCAGCTTTGTGATGCAGATTTTTCAC-----GTTGTT-ACCTCCA

[EC_hmp](http://ergo.integratedgenomics.com/ERGO/CGI/prot.cgi?prot=REC02492&user=rodionov:40164470) AATAAAGAGCTTG-CAACCGGAAACGGATTTCTTTTCAGGTTTGTGATGCAAATTTTTCAC-----TTCATC-ACATTCT

[KP_hmp](http://ergo.integratedgenomics.com/ERGO/CGI/prot.cgi?prot=RKP06691&user=rodionov:40164470) AATAACGCCTCTCTC-AGTATCAACGGATTTCTTTTCAGGTTTGTGATGCAAATTCTTCAT-----GTTGTA-ACTGGAT

ER_hmp TCTTTCCGGGATTAGCCATTCCGGCTGATTTTTTCTCATCCTCAACCTGCATTTTTTTCATACGCCTTTCCC-CCTGATT

EO_hmp AATATAAATCGCGTGGCGG-CAAGCAGATTTTTCTTCATTCTCAACCTGCTATTTCTTCAT--GTTATTCCC-TCAGGCT

YE_hmp TGTTCAGACTTCATCCAAG-GCAACGGAATTATCCTCATATTCATTATGTGATTTTTTCACATTCTGTGACCTGACACCG

YP_hmp TGTTCAGACTTCATTCAAT-GCAACAGAATTATCCTCATATTCACTATGTGAATTTTTCATCTTCAGCGA---GTAAGTG

* ** ** * *** * ** ** ****

[ST_hmp](http://ergo.integratedgenomics.com/ERGO/CGI/prot.cgi?prot=RTY05232&user=rodionov:40164470) TAACGTAAAGCAGAG----AAGATCCA---------TTTACAATGCAAGGGTATTTTT-ATAAGATGCATTTGATATACA

[EC_hmp](http://ergo.integratedgenomics.com/ERGO/CGI/prot.cgi?prot=REC02492&user=rodionov:40164470) TTCTGAAAAACACCA----AAGAACCA---------TTTACATTGCAGGGCTATTTTTTATAAGATGCATTTG**A**GATACA

[KP_hmp](http://ergo.integratedgenomics.com/ERGO/CGI/prot.cgi?prot=RKP06691&user=rodionov:40164470) CGGGGGAATGTGTTA----AAGAACCA---------TTTACAACGCAGGGTTATTTCT-TTAAGATGCATTTAAAATGCA

ER_hmp CGTTGCCCCGCCCCTACCCCAGACCATCACCGGGGATTTACAG-GCAACATCCTTTTTAATAAGATGCATTTAAAATGCA

[EO_hmp](http://ergo.integratedgenomics.com/ERGO/CGI/prot.cgi?prot=REO02801&user=rodionov:40164470) CACTCTACAAACCC---CCACCACCAA-AAAGGTAATTTACAG-CGACGGACAAAAACAATAAGATTCATATAAAATACA

YE_hmp CGT-GTTA--ATTCCT---TATAAGAAGGATGCGCATTTACAAATTAGGCTGCAATCT-ATAAGATGCATTTAAAATACA

YP_hmp CGTTGATACGATCCCCC--TACAGGTGAAAATTACATTTACAAAGAGAAGAGAAAACC-ATAAGATGCATTTAAAATACA

* ****** ****** *** * * ** **

[ST_hmp](http://ergo.integratedgenomics.com/ERGO/CGI/prot.cgi?prot=RTY05232&user=rodionov:40164470) TT---------ATTAGATTTTC----------------------------------------ACATAAAGGAAGC-ACG-

[EC_hmp](http://ergo.integratedgenomics.com/ERGO/CGI/prot.cgi?prot=REC02492&user=rodionov:40164470) TC---------AATTAAGATGC----------------------------------------AAAAAAAGGAAGA-CCA-

[KP_hmp](http://ergo.integratedgenomics.com/ERGO/CGI/prot.cgi?prot=RKP06691&user=rodionov:40164470) TC---------ATTAAGATAAC----------------------------------------ACATAA-GGAAGC-ACGC

ER_hmp AC-------TTATAGA----------------------------------------------CAACAA-TGAGGT-TCAC

[EO_hmp](http://ergo.integratedgenomics.com/ERGO/CGI/prot.cgi?prot=REO02801&user=rodionov:40164470) AC-------TTATAAAATATAC----------------------------------------CAATAAATAAGGAGTCAC

YE_hmp TGTATTGAATCACACACAATAACACCTGAAGTCATTGGCGTTGCAGTCAGGGCGAAAGGGATAAATATTTAAGGAGCACC

YP_hmp TGTATTAATTTGTATATTATTA----------------------------------------AAACACTTAAGGAGCCAC

* * * *

[ST_hmp](http://ergo.integratedgenomics.com/ERGO/CGI/prot.cgi?prot=RTY05232&user=rodionov:40164470) T-**ATG**CTTGACGCACAAACCATCGCTACAGTAAAGGCCACCATTCCCCTGCTGGTTGAAACAGGACCGAAACTGACCGCC

[EC_hm](http://ergo.integratedgenomics.com/ERGO/CGI/prot.cgi?prot=REC02492&user=rodionov:40164470) T-**ATG**CTTGACGCTCAAACCATCGCTACAGTAAAAGCCACCATCCCTTTACTGGTGGAAACGGGGCCAAAGTTAACCGCC

[KP_hmp](http://ergo.integratedgenomics.com/ERGO/CGI/prot.cgi?prot=RKP06691&user=rodionov:40164470) T-**ATG**CTTGACGCTCAAACCATCGCCACGGTAAAAGCCACCATTCCCCTGCTGGTAGAGACCGGCCCGAAATTAACCGCC

ER_hmp C-**ATG**CTGGATCAACAAACCATCGCCACCATCAAATCCACCATTCCTCTGCTGGCCGAAACCGGTCCGGCGTTGACCGCT

EO_hmp C-**ATG**CTGGATAACCACACTATCGCCATCGTTAAATCGACCATCCCTCTGCTGGCGGAAACCGGCCCGAAACTAACCGCA

[YE_hmp](http://ergo.integratedgenomics.com/ERGO/CGI/prot.cgi?prot=RYE01462&user=rodionov:40164470) GC**ATG**CTGGATAGTCAAACCATCGCCACCGTTAAATCCACTATTCCGTTACTGGCCGCCACTGGCCCCAAGCTAACGGCA

YP_hmp T-**ATG**CTGGATACCCAAACCATCGCTATCGTTAAATCTACCATCCCCTTGCTGGCTGCCACCGGCCCCAAACTGACAGCC

***** ** ** ** ***** * * ** * ** ** ** * **** * ** ** ** * ** **
