# Supplementary material for: Dissimilatory Metabolism of Nitrogen Oxides in Bacteria: Comparative Reconstruction of Transcriptional Networks
Source: PLoS Comput Biol. 2005 Oct 28;1(5):e55. doi: 10.1371/journal.pcbi.0010055 (PMC1274295; doi:10.1371/journal.pcbi.0010055)
Supplement: Figure S4 — (24 KB DOC) [file pcbi.0010055.sg004.doc]

**Supplementary Figure S4.** Multiple sequence alignment of the upstream regions of the *hcp-hcr* operons from *Vibrio* species. Genome abbreviations are listed in Table 2. Candidate NorR binding sites and sigma-54 promoters are highlighted in blue and green, respectively. The *hcp* start codons are in bold.

VFI TAGGCATTTCAAATTGATACAAACAAACCAAATGATGTATTTATAACATCATGCGCCGTA

PPr TAG---TATAAAATTAATATAAACGCACT---TGATGTAATTATCACATCAAGTG-CGTT

VP ----------AAATAACCAACCGCA-AGAAGCCGATGTAATTAAGACATCATTTG-CGTT

VV ----------GCGTAGGCAACTGAGTATTTCTTGATGTAAAAAAGACACGGGTA--GCGT

* * * ****** * ***

VFI GTCATTAATACATCAATAAGCAAAAAATGAAACAATAAAGACAT-ATAAATCAATAACTT

Ppr GTAATAACCACATCAATCGTTATACCAATAAAAAGTAAAGTTGTTAAAAATCAACAAGAT

VP GTCTTAATTACATCACCTCCTCTATCAAAAACAAACAAAGCCATTAAAAATCAACAAGAT

VV GTCTTTTTTACATCACAGGCATGAGTAATTTAAAAATAAATTCTTTAATTTCATATAGAT

** * ****** * * * ** * * *** * *

VFI AAAAA-TGGCACGCACTGTGCAATAGATGGTTCATCAATGTCGATTCAAATCTCGACTGG

Ppr AAAAGCTGGCACGTTAAATGCTAT---TAGTTC-----------TCCAAATCATCGTTAA

VP AATAATTGGCACGCATTCTGCT-----------------------------CTCTAGTGG

VV AACAGTTGGCACGCAATCTGCT-----------------------------CTTAGGTGA

** * ******* *** * *

VFI TTCAACAATCTATTGCACAATAGAACGCATTGAACTGGCATGATTTGAACAATATGTATT

Ppr TTCGATATT--------------------------------------AATAATATAAT--

VP --------TCAACATCACGACT--AT----TAAAACA-----AT----------------

VV AC--GTTTTTAATCTAATAACA--ACAACCTAAACCC-----ATTTCGTTTAGGGGTAAC

*

VFI AATGACTGGAGTTCTAACT**ATG**TTCTGTATCCAATGTGAGCAAACGATCCAAACACCAAC

Ppr TATAAC-GGAGTCCTAA--**ATG**GTCTGTATTCAATGTGAGCAAACTGTCCAAACACCTAC

VP --CACTGGAGAACCCAA-T**ATG**TTCTGTATTCAATGTGAACAGACAATTCAAACCCCTGC

VV GACACCGGAGACGCCAACT**ATG**TTCTGTATTCAATGTGAACAGACGATTCAAACCCCAGC

* * ** *** ******* ******** ** ** * ***** ** *
